# Interleukin 6 and interleukin 17A serum levels and gene- polymorphisms in the development of early allograft rejection in living donor liver transplant recipients

**DOI:** 10.1038/s41598-024-71102-1

**Published:** 2024-09-17

**Authors:** Samah Mohammed Awad, Eman Helmy El Batanony, Shaimaa K. Elmahdy, Esraa Tawfik Allam, Sara Kamal Rizk, Ahmed B. Zaid, Mohammad Taha, Radwa H. Salem

**Affiliations:** 1 Clinical Microbiology and Immunology Department, National Liver Institute, Menoufia University, Shibin Elkom, 32511 Egypt; 2 Gastroenterology and Hepatology Department, National Liver Institute, Menoufia University, Shibin Elkom, 32511 Egypt; 3 Departments of Clinical Pathology, National Liver Institute, Menoufia University, Shibin Elkom, 32511 Egypt; 4 Biochemistry department, Faculty of Medicine, Menoufia University, Shibin Elkom, 32511 Egypt; 5 Hepatopancreatobiliary and Liver Transplant Surgery, National Liver Institute, Shibin Elkom, 32511 Egypt

**Keywords:** Interleukin 6, Interleukin 17A, Serum levels, Gene- polymorphisms, Early allograft rejection, Infections, Immunology, Cytokines, Interleukins, Genetics, Cytogenetics

## Abstract

The aim of this study is to evaluate the role of serum level of Interleukin 6(*IL-6*) and Interleukin 17 (*IL-17*) in liver transplantation outcome for living recipients, Analyze the relation between the gene polymorphism and the occurrence of rejection after liver transplantation and Study the relation between the gene polymorphism and the occurrence of different infectious complications. The study was conducted in March 2023 and included 60 healthy volunteers from the National Liver Institute (NLI) blood bank at Menoufia University and 120 live donation liver recipient patients at NLI. During one month of liver transplantation, the cytokine levels (IL-17, IL-6 proteins, IL-6 G-174C, and IL-17 A rs2275913 gene polymorphism) and CD4 levels for 60 patients of 120 live donation liver recipient patients whom early reject transplanted tissue and the same parameters were measured after 6 months follow up for non-reject group. The main finding of this study was that the post-transplant rejection group and the post-transplant non-rejection and control groups differed significantly in the genotype frequency (CC, CG, and GG) or alleles of IL-6 G-174C (*p* = 0.011). On the other hand IL-17A rs2275913 gene polymorphism and its alleles (*p* = 0.71) showed no statistically significant difference. We also observed that serum IL-17 levels, with 100% specificity and 100% sensitivity threshold, will be more sensitive and specific than serum IL-6 and CD4 count in differentiating post-transplant rejection from non-rejection patients. The results showed that there was no significant relationship between the genotypes and serum levels of interleukins and the type and degree of rejection. Proinflammatory cytokines might be useful indicators for distinguishing and early identifying unfavorable outcomes after transplantation, allowing for prompt and effective treatment intervention. To evaluate these findings, prospective clinical trials are required.

## Introduction

For end-stage liver disease (ESLD), liver transplantation (LT) is regarded the sole effective therapy and a life-saving procedure^1^.

Liver-based metabolic abnormalities, severe cirrhosis in chronic liver disease, hepatocellular carcinoma, and acute liver failure are among the conditions that can be treated with a liver transplant^2^.

Even with all the positive developments in surgery and medication for liver transplantation, early allograft malfunction, viral infection recurrence, and inflammatory immune responses are still regarded as barriers to a successful procedure^3^.

One of the most significant immunological side effects after liver transplantation is graft rejection^4^, which is defined as increased transaminase and/or bilirubin, indicative of graft dysfunction, that initially remains elevated for a minimum of three times the upper normal limit without the presence of infection or vascular or biliary problems^5^. The transplanted tissue’s biopsy investigation reveals that host T cells and other mononuclear leukocytes have infiltrated the tissue, harming the graft^6^.

The liver recipient is now vulnerable to both the recurrence of latent infections that were previously dormant and de novo infections due to systemic immunosuppression. The most common cause of infection after LT is bacterial infections, which are most common in the first month following surgery because of invasive device usage, immunosuppression, and mucocutaneous barrier modification^7^.

Cytokines are biological messengers that are soluble chemicals that are involved in both pathologic and physiological immune responses, such as graft rejection, autoimmunity, allergies, and defence against infections^3^.

Cytokines may have an impact on immunosuppression or the outcome of immunosuppressive treatment utilised during organ transplantation. A person’s ability to produce a complex network of cytokines, growth factors, or adhesion molecules after graft transplantation plays a major genetic role in this process. These molecules can either mediate the graft acceptance or modulate the immune response towards rejection^8^.

Regarding graft rejection, there may be an up-, down-, or no change in the expression of the T helper 2 cytokine IL-6. IL-6 is a crucial mediator of the acute phase responses as well as an important regulator of inflammation. It has been discovered that following transplantation, blood levels of IL-6 rise noticeably during the hepatic phase and once again during the reperfusion phase^8^.

Th17 cells and their key cytokine, IL-17, are implicated in a number of inflammatory and autoimmune disorders, including multiple sclerosis, rheumatoid arthritis, psoriasis, and inflammatory bowel disease, as well as allograft rejection. Through its ability to induce the production of cytokines such as TNF-α, IL-4, IL-6, IL-18, and chemokine ligand, IL-17 plays protective and regulatory functions in immune response^9^. Increased IL-17 following transplant and rejection have been documented in many transplant types in earlier investigations^10^.

To our knowledge, the relation between IL-6 and IL-17A and adverse events following living donor liver transplantation is not well studied. So the aim of this study is to evaluate the role of serum level of Interleukin 6(*IL-6*) and Interleukin 17 (*IL-17*) in liver transplantation outcome for living recipients, Analyze the relation between the gene polymorphism and the occurrence of rejection after liver transplantation and Study the relation between the gene polymorphism and the occurrence of different infectious complications.

## Methods

### Study design

Our retrospective single-center investigation was conducted in March 2023 and approved by the Institutional Review Board (0046/2023) of the National Liver Institute (NLI) at Menoufia University in Egypt. It was conducted in accordance with the Guidelines for Good Clinical Practice and the Declaration of Helsinki. All participants gave their signed, informed consent after being made aware of the objectives and design of the study.

### Patients protocol Settings by the NLI hospital during observation period (hospital guidelines).

The time frame from the day before the conditioning regimen began to the day of release following liver transplantation (LT) was referred to as the observation phase. Standard prophylactic treatment, such as metronidazole, virostatics, anti-bacterials, and anti-mycotics, was administered to all participating patients who were transplant recipients. Sixty-one of the 120 patients in our research did not have any post-transplant problems, including sepsis, invasive or localized bacterial, viral, or fungal infections, veno-occlusive disease (VOD), acute Graft versus host disease (GvHD), or any other type of post-transplant complications. During one month of liver transplantation, the cytokine levels (IL-17, IL-6 proteins, IL-6 G-174C, and IL-17 A rs2275913 gene polymorphism) and CD4 levels for 60 patients of 120 live donation liver recipient patients whom early reject transplanted tissue and the same parameters were measured after 6 months follow up for non-reject group.

### Immunosuppression

According to the LDLT immunosuppression protocol^11^, patients who were scheduled for living donor liver transplantation (LDLT) were given an immunosuppression regimen that included prednisolone, mycophenolate mofetil (MMF), calcineurin inhibitors (such as tacrolimus or cyclosporine), and basiliximab (IL-2 receptor blocker). For the first month following LDLT, the tacrolimus trough level was maintained between 7 and 10 ng/mL; it was then lowered to 5–7 ng/mL. For the first month following LDLT, the cyclosporine trough level was maintained at 100–150 ng/mL, and then it serially dropped to 50–100 ng/mL. Following LDLT, prednisolone was gradually discontinued during the first month, while MMF was tapered three to six months following surgery. On the day before to LDLT surgery as well as on postoperative day (POD) 4, basiliximab was given.

### Criteria for the assessment of post-transplant adverse events

In order to grade liver transplant rejection, the diagnosis will be made in accordance with the Banff Schema^12^. Rejection that occurs in the initial months following transplantation will be referred to as allograft rejection^12^. The International Sepsis Consensus Conference on Critical Care 2005 criteria were used to evaluate sepsis^13^. Bacteremia was diagnosed based on at least one positive blood culture. A positive polymerase chain reaction test for any of the following viruses: parvovirus B19, human herpes simplex virus (HSV), varicella zoster virus (VZV), HHV-6, Epstein-Barr virus (EBV), Cyto-Megalo Virus (CMV), and adenovirus (ADV) in blood was deemed to be viremia. A local bacterial and viral infection, often referred to as a noninvasive bacterial, viral, or fungal infection in the blood, was diagnosed based on the results of a microbiological or virological test for infection in the pharynx, urine, or faeces. The National Institute of Allergy and Infectious illnesses Mycoses Study Group (EORTC/MSG) and the European Organization for Research and Treatment of Cancer’s Invasive Fungal Infections Cooperative Group have developed criteria for invasive fungal illnesses were followed when defining proven or probable invasive fungal infections^14^.

### Postoperative acute cellular rejection in recipients

Elevations in liver enzyme values (total bilirubin, INR, AST, and ALT) within 90 days of LDLT were linked to clinical symptoms (fever and jaundice). The diagnosis of acute cellular rejection was made, and a liver biopsy was used to confirm it. A rejection activity index (RAI) of three points was used, calculated by experienced pathologists using the Banff system^12^.

### Studied groups

The study have been carried out on 120 living donor liver recipient patients in NLI and 60 healthy volunteers’ participant from blood bank of NLI, Menoufia University. All patients and control group ages ranged between 18 and 76 years old.

1. Sixty patients with allograft rejection
2. Sixty Patients without allograft rejection
3. Sixty healthy volunteers participant from National Liver Institute blood bank

## Materials

### Sampling & preservations

This retrospective single-center investigation comprises a longitudinal analysis of cytokine levels IL-17, IL-6 proteins & *IL-6 G-174C and IL-17 A rs2275913* gene polymorphism, flow- cytometry analysis for CD4Tcells and routine hematological & biochemical analysis of consecutive adult patients before and after liver transplantation. A sterile procedure was used to draw blood into test tubes (BD Vacutainer, K2 EDTA; Becton, Dickinson and Company, Franklin Lakes, NJ, USA); these test tubes were intended for use in serological analysis. All blood samples—aside from blood cultures, which were processed right away—were brought to the lab in an ice-filled box, centrifuged for 10 min at 4500 rpm at 4°C, frozen at − 70°C, and kept in storage until a separate assay was performed.

### Different techniques used to operate different analysis

#### Diagnosis of infections

- **VITEK®** **2 COMPACT** automated microbial identification and antimicrobial sensitivity system (Biomerieux) for Bacterial identification and antimicrobial sensitivity. BacT/ALERT® automated microbial detection system (Biomerieux) for Blood culture.
- **Chromogenic media (CONDA)** for fungal infection identification.
- **Real time –PCR** for diagnosis of (CMV), (HBV), (HCV), (ADV),(EBV), (VZV), (HSV), B19 virus .
- **Sandwich enzyme-linked immunosorbent assays**:
- Human IL-17A, Biosource, and USA, as well as Human IL-6, and Biosource, USA, were used to assess the levels of serum cytokines. The LuminexTM detection system (200TM; Luminex Corp., Austin, TX, USA) was used to evaluate the data.
- **Flow-cytometry To assess serum CD4-TLymphocytes:**
- Process The CD4 T cell subset was evaluated using FACS Calibur flow cytometry (BD Bio-sciences, San José, CA, USA). MAb against CD4 APC (Article Processing Charge) was used to identify the cells (UCHT1). In 12 × 75 test tubes, 100 ul of whole EDTA blood was combined with 20 ul of each monoclonal reagent. The tubes were then allowed to sit at room temperature for 15 min while being kept in the dark. After adding one milliliter of NH4Cl (0.83% buffered with KHCO3, pH 7.2) and centrifuging the mixture for five minutes at 2000 rpm, the erythrocytes were lysed. After discarding the supernatant, the cells underwent two rounds of washing in phosphate-buffered saline (PBS) and were then resuspended in 300 ul of PBS. FACS Calibur and Flowjo software (Tree Star, Ashland, OR USA) were used to analyses the data. To define nonspecific fluorescence, negative isotope matched controls (Coulter Beckman, USA) were performed. For lymphocyte gating, standard forward and side scatters were employed. Selecting the CD4-population further gated the already gated lymphocytes.
- **C6000 auto analyzer, Roche diagnostics, Germany (Roche diagnostics- GmbH, D-68305 Mannheim, (Germany)** for assessment Liver function tests including Serum Albumin (gm /dl), ALT(IU/L), AST(IU/L) & Total Bilirubin(mg/dl) tests were carried out using
- **Practical enhanced immunoturbidimetric assay** was used to measure the levels of **C-reactive protein (CRP):** Latex particles coated with monoclonal anti-CRP antibodies agglutinate human CRP. Turbidity metrics are used to determine the aggregates^15^**.**
- **Automatic cell counter; Sysmex XT 1800 (Germany):** for assessment complete blood counts; Hemoglobin level (Hb) (gm/dl), White blood cells count (WBCs) (cell/mcl). Platelets count (PLTs) (plt/mcl) Complete blood picture was done on
- ***DNA extraction, SNP primers design, and genotyping For IL6 G-174C***
- **DNA extraction:** Thermo Scientific Gene JET Genomic DNA Purification Kit (cat#K0721, Lithuania) was used to extract DNA from whole blood according to manufacturer’s instructions. The DNA that was gathered had its purity and quantity validated by Nano drop. DNA samples were taken out and kept for future use at − 20°C In order to genotype the IL-17 rs2275913 SNP, the amplification refractory mutation system (ARMS) polymerase chain reaction was employed. Allele-specific primers were generated using the NCBI-GenBank database and Batch Primer design online using an online SNP tool. The primer sequences that were utilised include Common Reverse5′-GAGTAGTTTCCGGAATTGT-3′, Allele G-specific forward 5′-GAGGTCATAGAAGAATCTCAC -3′, and Allele A-specific forward 5′-GAGGTCATAGAAGAATCTCTT-3′. The primers used in the investigation were bought from Macrogen in Korea. Initial denaturation (at 95°C for 4 min) was followed by 25 time cycles of denaturation (30 s at 95°C, annealing (30 s at 53°C), extension (30 s at 72°C each cycle), and final extension (7 min at 72°C). On a gel of 2% agarose, to ascertain the genotypes for rs2275913, the existence or lack of a PCR amplicon (320 bp) corresponding to the specific allele (A/G) was utilised.

The technique employed was the mutagenically separated polymerase chain reaction (MS-PCR). based on the study of Schotte et al.^16^**,** Three different combinations of primers were used. The forward primer (G) is 5′-GCACTTTTCCCCCTAGTTGTGTCTTACG-3′; the reverse primer is 5′-ATAAATCTTTGTTGGAGGGTGAGG-3′; the forward primer (C) is 5′-GACGACCTAAGCTTTACTTTTCCCCCTAGTTGTGTCTTGAC-3′.Aswas previously noted, the reaction was carried out in a single tube with a 25 ul final reaction volume^17^**.** 40 ng of DNA, 10 Pmoles of each forward primer (Primer G) and reverse primer (Primer C), and Dream Taq Green PCR Master Mix (2X) (Fermentas) were used. A Biometra heat cycler (Biometra GmbH, Germany) was used for all reactions. The following were the PCR cycling conditions: For this experiment, we used 40 ng of DNA, 10 Pmoles of each forward primer (Primer G) and reverse primer (Primer C), and Dream Taq Green PCR Master Mix (2X) (Fermentas). Biometra GmbH, Germany manufactured the heat cycler that was used for all of the processes. The PCR cycle conditions were as follows: After 1 cycle at 95 °C for 10 min, 40 cycles at 94 °C for 30 s, 66 °C for 45 s, and 72 °C for 45 s, and 1 cycle at 72 °C for 7 min. For the G allele, the PCR results were 121 bp, and for the C allele, 136 bp.

### Statistical analysis

G. Power version 3.1.9.4, a free sample size calculator, was used to determine the sample size. With an effect size of 0.25, a power of 85%, and a statistical significance threshold of 0.05. Cohen’s calculation yielded a sample size of 180 people, which is also the number of participants in the current study.

Data were loaded into the computer, and analysis was performed using IBM SPSS software version 20.0 (IBM Corp, Armonk, NY). Utilizing percentages and numbers, the qualitative data was described. The chi-square test was employed to compare the two groups. In contrast, the Monte Carlo adjustment test was used if the expected cell counts were less than 5. The normality of continuous data was examined using the Kolmogorov–Smirnov test. Distributed data were described using the phrases mean, standard deviation, median, and range (minimum and maximum). The student t-test was used to compare two groups for normally distributed quantitative variables; an ANOVA was used for the four research groups; and the Post Hoc test (Tukey) was used for pairwise comparisons. As though the Kruskal Wallis test was employed to contrast several groups for non-normal distributed quantitative variables and followed by Post Hoc tests (Dunn’s for multiple comparisons test) for pairwise comparison. Significance of the obtained results was judged at the 5% level.

## Results

### Characteristics of the study population

For our study, one hundred and eighty people were enrolled from the national liver institute’s inpatient gastroenterology clinic at Menoufia University between January 5, 2023, and November 1, 2023. Of these, 60 patients had liver transplant rejection and were included in group (1); the remaining 60 patients underwent liver transplantation but did not experience rejection were randomly assigned to group (2). Group (3) comprised sixty healthy volunteers from the NLI blood bank. Demographic, clinical and laboratory data for our study were summarized in Tables 1, 2, 3**, **Fig. 3**.**

**Table 1:** Baseline demographic and biochemical parameters for the three studied groups.

	Reject group (n = 60)	Non-reject group (n = 60)	Control (n = 60)	Test of Sig	*p*	Sig. bet. grps		
						1 versus 2	1 versus 3	2 versus 3
Age (years)Mean ± SD	46.7 ± 10.4	47.4 ± 10.1	44.3 ± 10.3	F = 1.499	0.226	> 0.05	> 0.05	> 0.05
Sex								
Male	52 (86.7%)	46 (76.7%)	52 (86.7%)	χ^2^ = 2.880	0.237	> 0.05	> 0.05	> 0.05
Female	8 (13.3%)	14 (23.3%)	8 (13.3%)					
Residence								
Urban	31 (51.7%)	26 (43.3%)	22 (36.7%)	χ^2^ = 2.752	0.253	> 0.05	> 0.05	> 0.05
Rural	29 (48.3%)	34 (56.7%)	38 (63.3%)					
T. Bilirubin (mg/dl) Median (Min.–Max.)	1.9 (1–36.6)	0.9 (0.2–1.4)	0.5 (0.2–0.9)	H = 140.769*	< 0.001*	< 0.001*	< 0.001*	< 0.001*
D. Bilirubin (mg/dl) Median (Min.**–**Max.)	1.1 (0.1–24.9)	0.4 (0.1–1)	0.2 (0–0.3)	H = 112.766*	< 0.001*	< 0.001*	< 0.001*	< 0.001*
AST(IU/L) Median (Min.**–**Max.)	201.5 (56–589)	75 (19–215)	24.5 (16–35)	H = 129.283*	< 0.001*	< 0.001*	< 0.001*	< 0.001*
ALT(IU/L) Median (Min.**–**Max.)	265 (48–680)	80.5 (23–319)	35 (22–41)	H = 125.279*	< 0.001*	< 0.001*	< 0.001*	< 0.001*
Total protein(g/dl) Median (Min.**–**Max.)	6.9 (5.3–8.6)	7.7 (6.4–8.5)	7.7 (6.5–8.4)	F = 15.420*	< 0.001*	< 0.001*	< 0.001*	0.491
S. Albumin (g/dl) Median (Min.**–**Max.)	3.5 (2.1–4.7)	3.9 (2.4–4.8)	4.2 (3.1–5.1)	H = 18.043*	< 0.001*	< 0.001*	< 0.001*	< 0.001*
ALK. phosph.(IU/L) Median (Min.**–**Max.)	596.5(120–3201)	135.5 (95–443)	86 (45–127)	H = 143.518*	< 0.001*	< 0.001*	< 0.001*	< 0.001*
GGT(IU/L) Median (Min.**–**Max.)	593.5(185–1980)	76 (52–517)	39 (21–65)	H = 148.828*	< 0.001*	< 0.001*	< 0.001*	< 0.001*
S. urea(mg/dl) Median (Min.**–**max.)	50 (23–99)	60.5 (34–87)	35 (16–53)	H = 71.885*	< 0.001*	0.051	< 0.001*	< 0.001*
S. creatinine(mg/dl) Median (Min.**–**Max.)	1.2 (0.8–2.3)	0.9 (0.5–1.4)	0.9 (0.7–1.4)	H = 44.313*	< 0.001*	< 0.001*	< 0.001*	0.212

*Statistically significant at *p* ≤ 0.05, Group1: Post-transplant rejection,Group2: Post-transplantation non rejection, Group3: Control.

**Table 2:** Baseline haematological and immunological parameters for the three studied gps.

	Reject group (n = 60)	Non-reject group (n = 60)	Control (n = 60)	Test of Sig	*p*	Sig. bet. Gps		
						1 versus 2	1 versus 3	2 versus 3
PT(sec) Median (Min.–Max.)	15 (12**–**22)	12.7 (11.7**–**14.6)	12.5 (11.2**–**13.4)	H = 73.400	< 0.001*	< 0.001*	< 0.001*	0.022*
HB (gm/dl) Mean ± SD	13.3 ± 1.4	12.9 ± 1.1	13.4 ± 1	F = 2.741	0.067	> 0.05	> 0.05	> 0.05
Platelets(× 1000/ul) Median (Min.–Max.)	145 (55**–**529)	197 (113**–**242)	234 (213**–**432)	H = 65.416*	< 0.001*	0.087	< 0.001*	< 0.001*
TLC/µl × 10 3 Median (Min.–Max.)	6.5 (2.7**–**18.1)	5.6 (2.2**–**9.5)	6.7 (5.6**–**7.8)	H = 6.989*	0.030*	0.062	0.489	0.011*
lymphocytes% Mean ± SD	26 ± 8.1	25.8 ± 5.3	31.1 ± 4.5	F = 14.408*	< 0.001*	0.976	< 0.001*	< 0.001*
Lymphocytes count Median (Min.–Max.)	1636 (378**–**4068)	1409.5(374–3118.5)	2184 (1425**–**2652)	H = 22.636*	< 0.001*	0.041*	0.007*	< 0.001*
CD4% Median (Min.–Max.)	7.9 (2.5**–**32)	18.9 (6**–**44)	46 (36**–**55)	H = 134.785*	< 0.001*	< 0.001*	< 0.001*	< 0.001*
CD4 count Median (Min.–Max.)	144.3(20.1**–**406.3)	296.5(78.5**–**691.3)	927.5(648**–**1310.4)	H = 127.636*	< 0.001*	0.002*	< 0.001*	< 0.001*
CRP(mg/dl) Median (Min.–Max.)	23.5 (4.8**–**102.6)	9.5 (5.6**–**48.8)	5.5 (4.7**–**6.2)	H = 100.861*	< 0.001*	0.020*	< 0.001*	< 0.001*
S. IL17(pg/ml) Median (Min.–Max.)	57.5(26.1**–**95.9)	8.4 (4.0**–**12.0)	3.8 (1.8**–**9.7)	142.045*	< 0.001*	< 0.001*	< 0.001*	< 0.001*
S. IL6(pg/ml) Median (Min.–Max.)	46.5(20.4–232.0)	8.6 (2.7**–**27.0)	4.0 (0.5**–**11.7)	129.636*	< 0.001*	< 0.001*	< 0.001*	0.001*

*Statistically significant at *p* ≤ 0.05, Group1: Post-transplant rejection, Group2: Post transplantation non rejection, Group3: Control.

**Table 3:** Baseline clinical data for the three studied groups.

	Reject group (n = 60)	Non-reject group (n = 60)	χ^2^	*p*
Clinical presentation				
Jaundice	40 (66.7%)	0 (0%)	60.0*	< 0.001*
Itching	14 (23.3%)	0 (0%)	15.849*	< 0.001*
Abdominal pain	30 (50%)	14 (23.3%)	9.187*	0.002*
Fever	34 (56.7%)	26 (43.3%)	2.133	0.144
Fatigue	4 (6.7%)	12 (20%)	4.615*	0.032*
Bone aches	6 (10%)	0 (0%)	6.316*	^FE^*p* = 0.027*
Diarrhea	12 (20%)	0 (0%)	13.333*	< 0.001*
Co morbidities				
Obesity	14 (23.3%)	16 (26.7%)	0.178	0.673
Hypertension	20 (33.3%)	22 (36.7%)	0.147	0.702
Renal disease	14 (23.3%)	8 (13.3%)	2.004	0.157
Diabetes	18 (30%)	24 (40%)	1.319	0.251
Family history				
HCV	38 (63.3%)	40 (66.7%)	0.147	0.702
HCC	22 (36.7%)	20 (33.3%)		
Original disease				
HCV, HCC	18 (30%)	22 (36.7%)	1.371	0.712
HCV	30 (50%)	26 (43.3%)		
HBV	6 (10%)	4 (6.7%)		
Others	6 (10%)	8 (13.3%)		
Donor degree				
First degree	22 (36.7%)	28 (46.7%)	1.474	0.479
Second	24 (40%)	22 (36.7%)		
Third degree	14 (23.3%)	10 (16.7%)		
Immunosuppressant regimen				
Tacrolimus	22 (36.7%)	36 (60%)	6.541*	0.011*
Steroids	60 (100%)	60 (100%)	–	–
MMF	28 (46.7%)	28 (46.7%)	0.00	1.000
Cyclosporine	38 (63.3%)	24 (40%)	6.541*	0.011*
Viral	0 (0%)	0 (0%)	–	–
Bacterial infection				
Negative	30 (50%)	34 (56.7%)	0.536	0.464
Positive	30 (50%)	26 (43.3%)		
Single or multiple bacterial infection				
Single	36 (60%)	41 (68.3%)	0.906	0.341
Multiple	24 (40%)	19 (31.7%)		
Site of infection				
UTI	14 (23.3%)	22 (36.7%)	2.540	0.111
SSI	12 (20%)	22 (36.7%)	4.104*	0.043*
Chest infection	32 (53.3%)	12 (20%)	14.354*	< 0.001*
BSI	28 (46.7%)	23 (38.3%)	0.853	0.356
Type of organism				
Staph aureus	28 (46.7%)	28 (46.7%)	0.00	1.000
Klebsiella	26 (43.3%)	27 (45%)	0.034	0.854
E.coli	6 (10%)	12 (20%)	2.353	0.125
Pseudomonas	10 (16.7%)	8 (13.3%)	0.261	0.609
Fungal infection				
Negative	42 (70%)	50 (83.3%)	2.954	^MC^*p* = 0.243
Candida throat	14 (23.3%)	8 (13.3%)		
Candida urine	4 (6.7%)	2 (3.3%)		
Rejection type and degree				
Mild acute rejection	24 (40%)	–	–	–
Moderate acute cellular rejection	28 (46.7%)	–		
Sever acute cellular rejection	8 (13.3%)	–		

*Statistically significant at *p* ≤ 0.05.

**Fig. 1:**
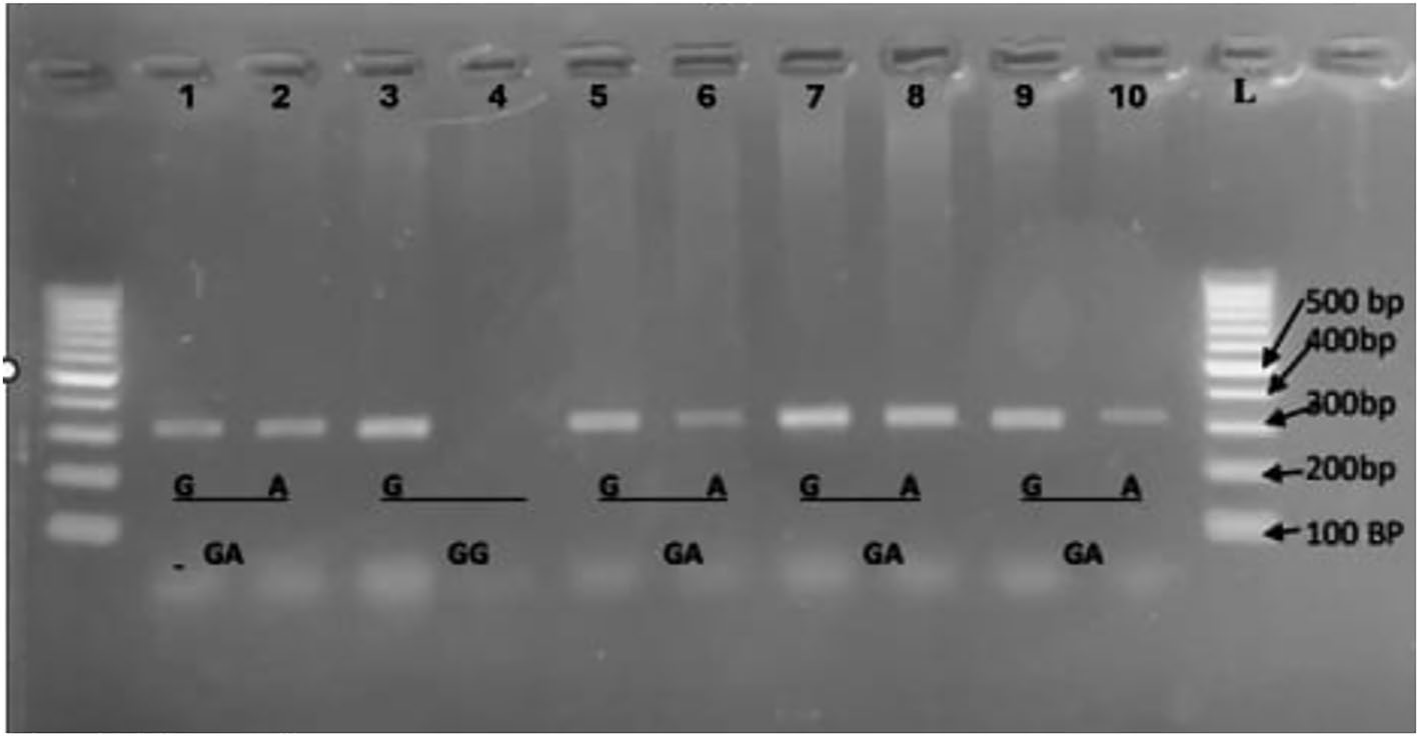
Amplification refractory mutation system-polymerase chain reaction of IL-17A rs2275913 genotyping PCR product. L: 100bp DNA ladder, lanes 1, 2, lanes 5, 6, lanes 7, 8 and lanes 9, 10 showed GA genotype whereas lane 3, 4 was GG genotype.

The genotypic and allelic frequencies of the IL6 G-174C and IL17A rs2275913 gene polymorphisms were determined in a number of adult patients after liver transplantation, along with control groups. The genotyping of the IL-6 G-174C and IL-17A rs2275913 gene polymorphisms was evaluated in adult patients who had received liver transplantation, as well as in controls, using two sets of allele-specific primers. As demonstrated in Fig. 1, the IL-17A rs2275913 gene polymorphism was amplified positively in both PCR reactions, resulting in a GG genotype; conversely, the first PCR reaction’s positive amplification only revealed a GA genotype; and the second PCR reaction’s positive amplification only revealed an AA genotype.

**Fig. 2:**
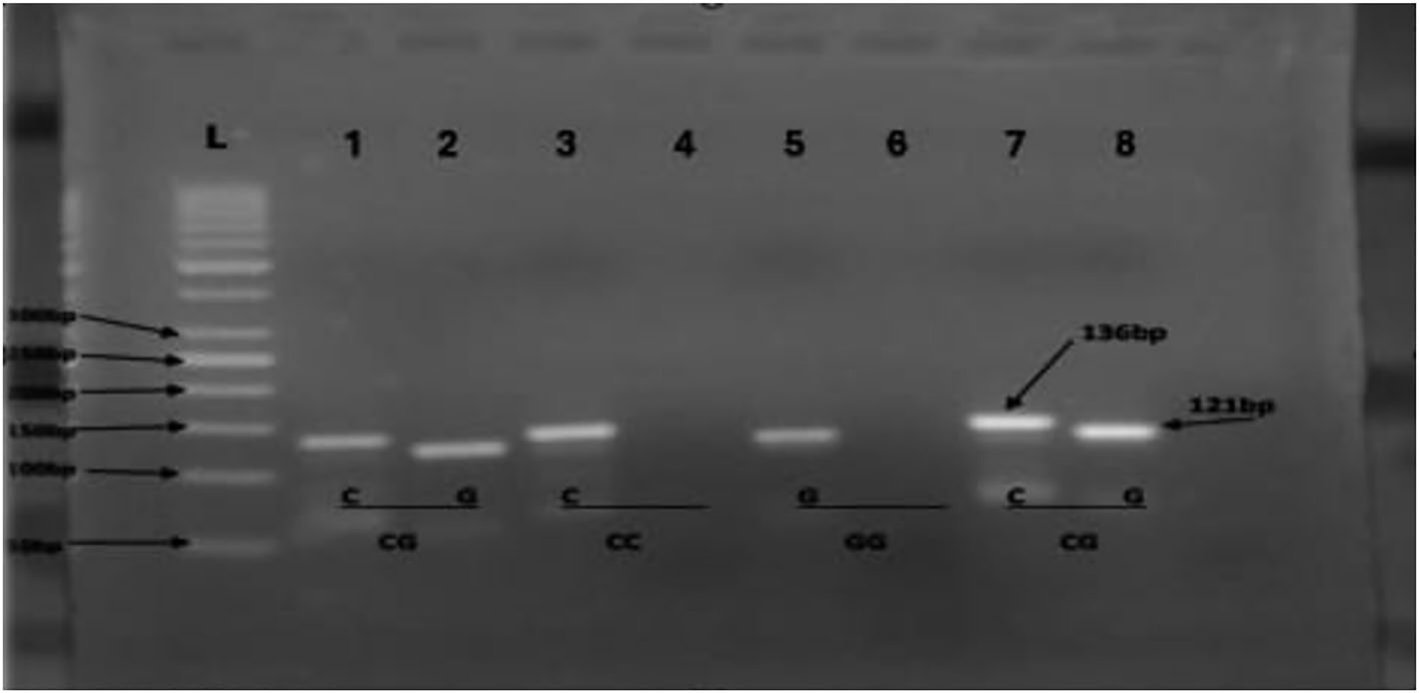
Amplification refractory mutation system-polymerase chain reaction of IL-6 (− 174 G/C) SNP analysis. Sample No.1 (lane1, 2), Sample No.4 (lane 7, 8) show CG genotype, Sample No.2 (lane 3, 4) shows CC genotype whereas Sample 3(lane 5,6) show GG genotype, L: Ladder DNA.

**Fig. 3:**
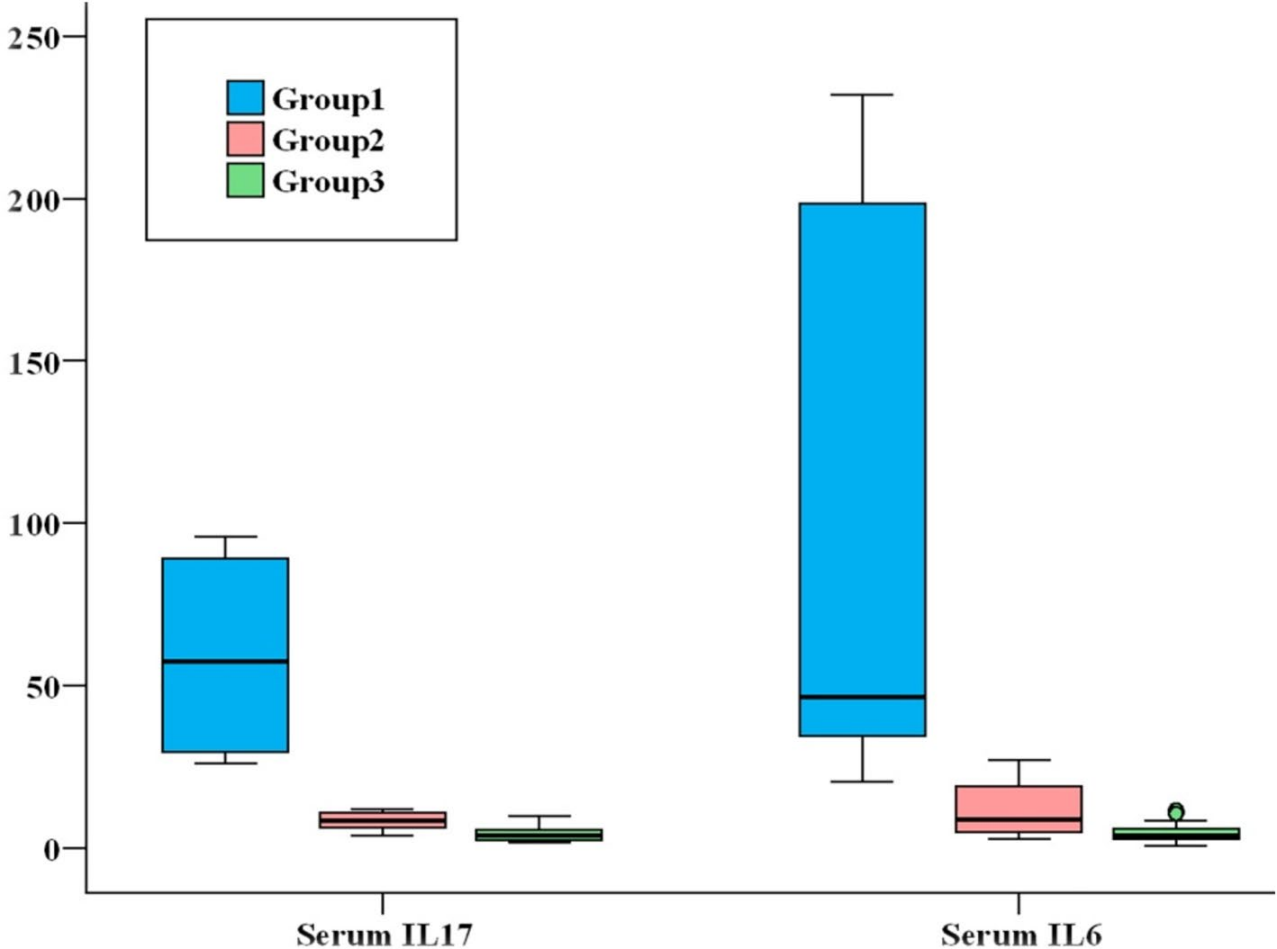
Comparison between the three studied groups according to Serum IL-17 and Serum IL-6.

Positive amplification in both PCR reactions indicated a CC genotype for the IL-6 G-174C gene polymorphism, while positive amplification in only the first PCR reaction revealed a CG genotype. Finally, the IL-6 G-174C gene polymorphism was examined. As shown in Fig. 2, the positive amplification in the second reaction only indicated a GG genotype. There was very little (*p* > 0.05) variation in the distribution of IL-17A genotypes at rs2275913, according to a test for Hardy–Weinberg (H–W) equilibrium. Table 4 shows that there was no significant difference (*p* > 0.05) in the frequency of any of the GG, GA, and AA genotypes or alleles of IL-17A at position rs2275913 between the patient groups and the control group. The post-transplant rejection group and each of the post-transplant non-rejection and control groups had significantly different genotypes (CC, CG, and GG) or alleles of IL-6 G-174C, but there was no significant difference between the genes and alleles of the post-transplant non-rejection and control group (*p* = 0.011) **(**Table 4**,** Fig. 3).

**Table 4:** Comparison between the three studied groups according to IL-17 gene and IL-6 gene.

	Reject group (n = 60)	Non-reject group (n = 60)	Control (n = 60)	*p*	Sig. bet. grps		
					1 versus 2	1 versus 3	2 versus 3
IL-17 gene							
GG	9(15%)	10(16.7%)	17(28.3%)	0.188	> 0.05	> 0.05	> 0.05
GA	36(60%)	37(61.7%)	36(60%)				
AA	15(25%)	13(21.7%)	7(11.7%)				
^HW^p_0_	**0.100**	**0.067**	**0.070**				
Allele							
G	54(45%)	57(47.5%)	70(58.3%)	0.090	> 0.05	> 0.05	> 0.05
A	66(55%)	63(52.5%)	50(41.7%)				
IL6 gene							
CC	2(3.3%)	10 (16.7%)	12(20%)	^MC^*p* = 0.031*	0.016*	0.011*	0.760
CG	31(51.7%)	34 (56.7%)	30(50%)				
GG	27(45%)	16 (26.7%)	18(30%)				
^HW^p_0_	**0.052**	**0.262**	**0.937**				
Allele							
C	35(29.2%)	54(45%)	54(45%)	0.015*	0.011*	0.011*	1.000
G	85(70.8%)	66(55%)	66(55%)				

*Statistically significant at *p* ≤ 0.05, Group1: Post-transplant rejection, Group2: Post transplantation non rejectionGroup3: Control.

Significant values are in bold.

No significant differences were observed in the frequency of IL-17A genotypes (GG, GA, and AA) between the patient and control groups (*p* = 0.416) for bacterial infection. However, there were significant differences in the frequency of IL-6 G-174C genotypes (CC, CG, and GG) between the patient and control groups (p < 0.001), indicating that infection may be a contributing factor (Table 5**).**

**Table 5:** Relation between bacterial infection and fungal infection with Serum IL-17, Serum IL-6 and IL-17 gene, IL-6 gene (n = 120).

	Bacterial infection		Fungal infection	
	Negative (n = 64)	Positive (n = 56)	Negative (n = 92)	Positive (n = 28)
IL17 gene				
GG	10 (15.6%)	9 (16.1%)	18 (19.6%)	1 (3.6%)
GA	42 (65.6%)	31 (55.4%)	59 (64.1%)	14 (50.0%)
AA	12 (18.8%)	16 (28.6%)	15 (16.3%)	13 (46.4%)
(*p*)	**(0.416)**		(0.002*)	
IL6 gene				
CC	10 (15.6%)	2 (3.6%)	12 (13.0%)	0 (0.0%)
CG	42 (65.6%)	23 (41.1%)	53 (57.6%)	12 (42.9%)
GG	12 (18.8%)	31 (55.4%)	27 (29.3%)	16 (57.1%)
(*p*)	(< 0.001*)		(0.010*)	

*Statistically significant at *p* ≤ 0.05.

Significant values are in bold.

As regarded fungal infection, the genotypes (GG, GA, and AA) frequency of IL-17A and The genotypes (CC, CG, and GG) frequency of *IL-6 G-174C* showed significant differences between patient groups and control group *p* = 0.002,0.01respectively **(**Table 5**).**

**Fig. 4:**
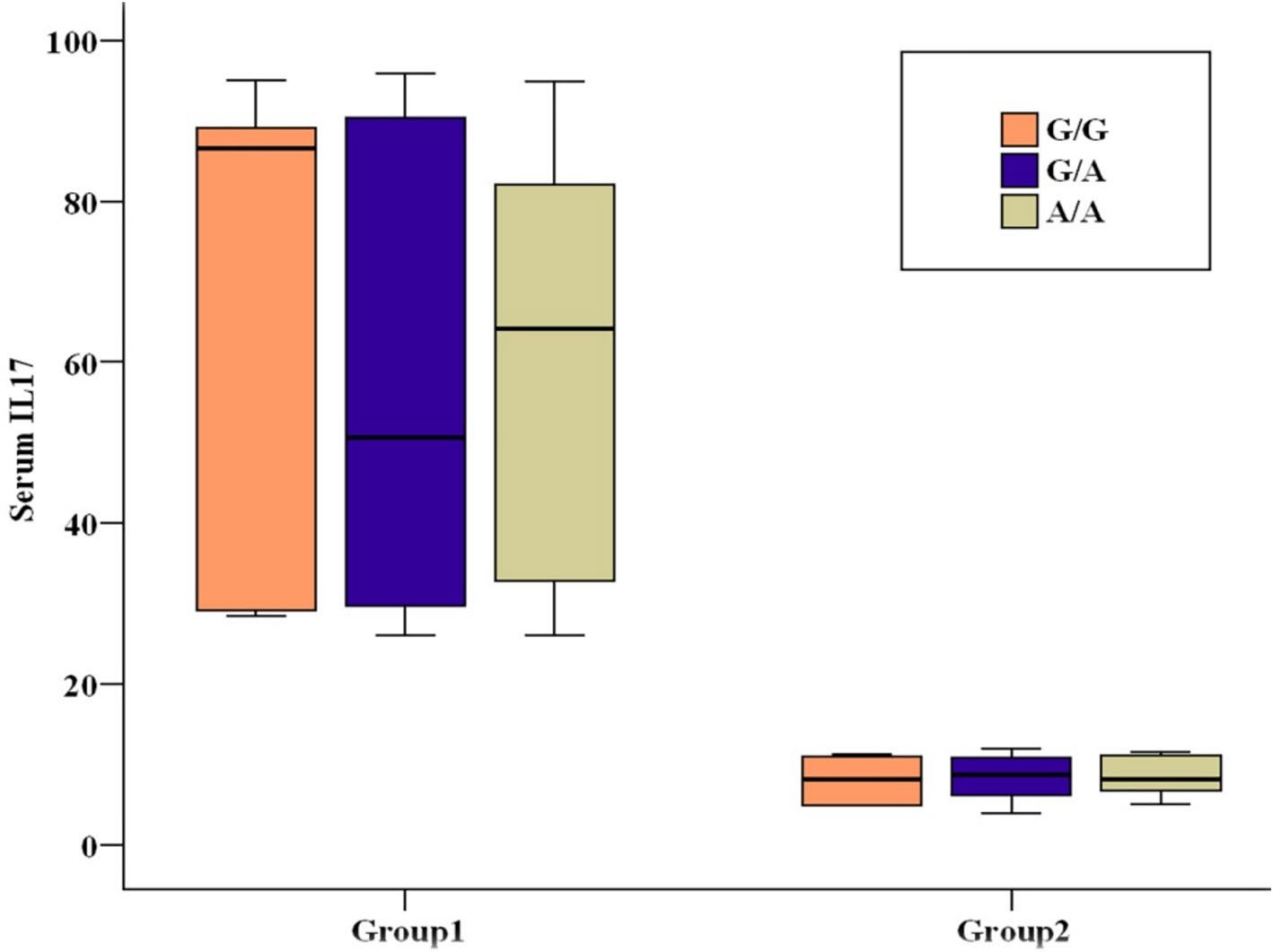
Relation between IL-17 gene and Serum IL-17 in each group.

**Fig. 5:**
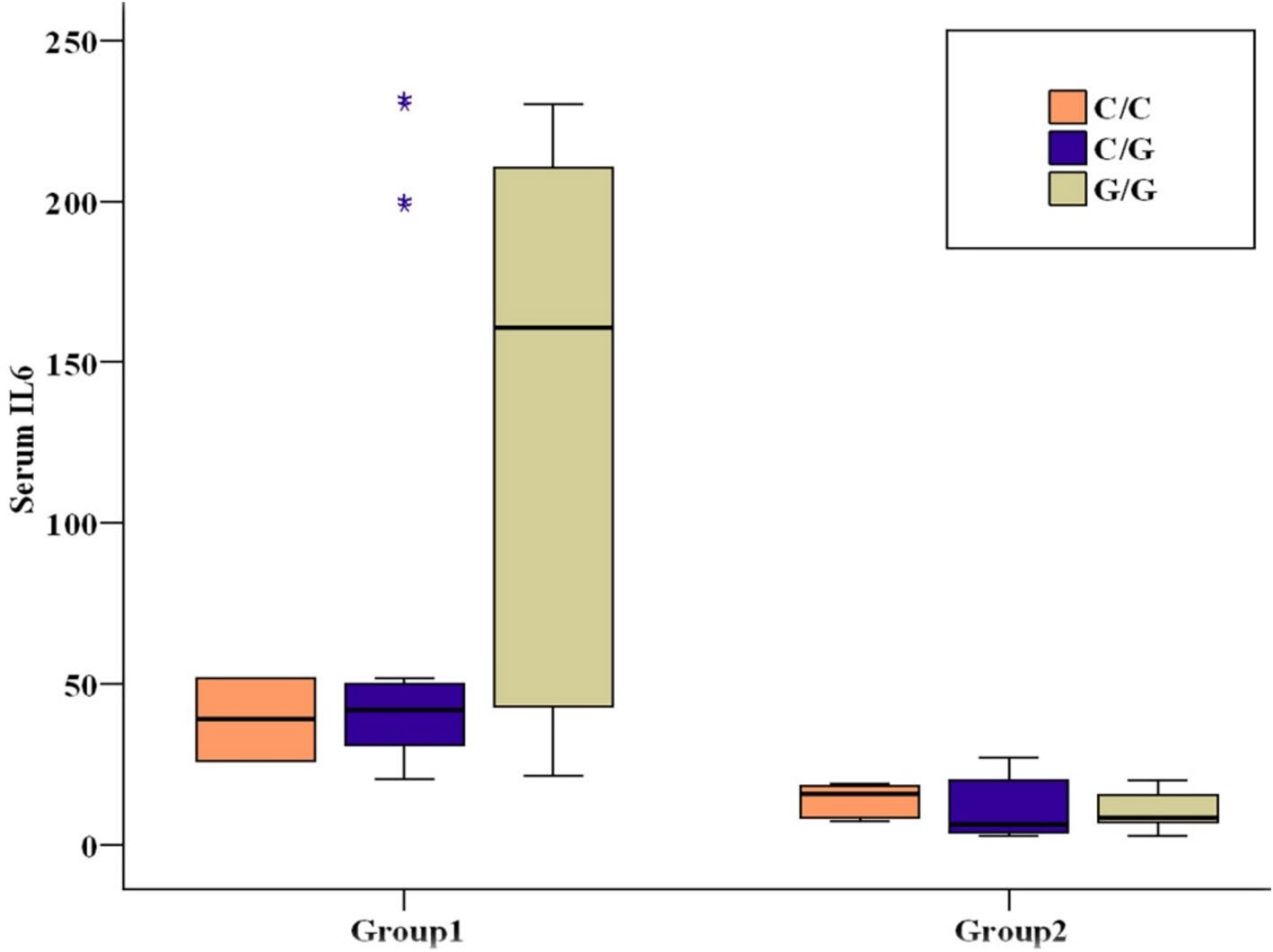
Relation between IL-6 gene and Serum IL-6 in each group.

To enhance comprehension of the function of interleukins gene polymorphisms in transplant post-rejection, the effects of interleukins genotypes on the production of interleukins under investigation were ascertained and distributed based on the patients’ serum level in each of the three genotypes in two sets of patients. The two patient groups with different genotypes displayed significant differences in serum levels of IL-17A and IL-6 **(**Figs. 4 and 5**).**

We used the Kruskal Wallis and Chi square test to examine the relationship between the type and degree of rejection with serum IL-17, serum IL6, and the IL17 and IL-6 genes in Group 1. The results showed that there was no significant relationship between the genotypes and serum levels of interleukins and the type and degree of rejection (Table 6**)**.

**Table 6:** Relation between Rejection type and degree with Serum IL-17, Serum IL-6 and IL-17 gene, IL-6 gene in Group 1 (n = 60).

	Rejection type and degree			*p*
	Mild acute rejection (n = 24)	Moderate acute cellular rejection (n = 28)	Sever acute cellular rejection (n = 8)	
Serum IL-17(pg/ml)				
Median (Min.–Max.)	69 (26.8–95.9)	61.1(26.1–94.9)	56 (29.8–75.9)	
Serum IL-6(pg/ml)				
Median (Min.–Max.)	71 (25–230.1)	42.3 (20.4–232)	44 (34.6–176.7)	
IL-17 gene				
GG	5 (20.8%)	4 (14.3%)	0 (0.0%)	^MC^*p* = 0.088
GA	17 (70.8%)	15 (53.6%)	4 (50.0%)	
AA	2 (8.3%)	9 (32.1%)	4 (50.0%)	
IL-6 gene				
CC	0 (0.0%)	2 (7.1%)	0 (0.0%)	^MC^*p* = 0.111
CG	10 (41.7%)	18 (64.3%)	3 (37.5%)	
GG	14 (58.3%)	8 (28.6%)	5 (62.5%)	

**Fig. 6:**
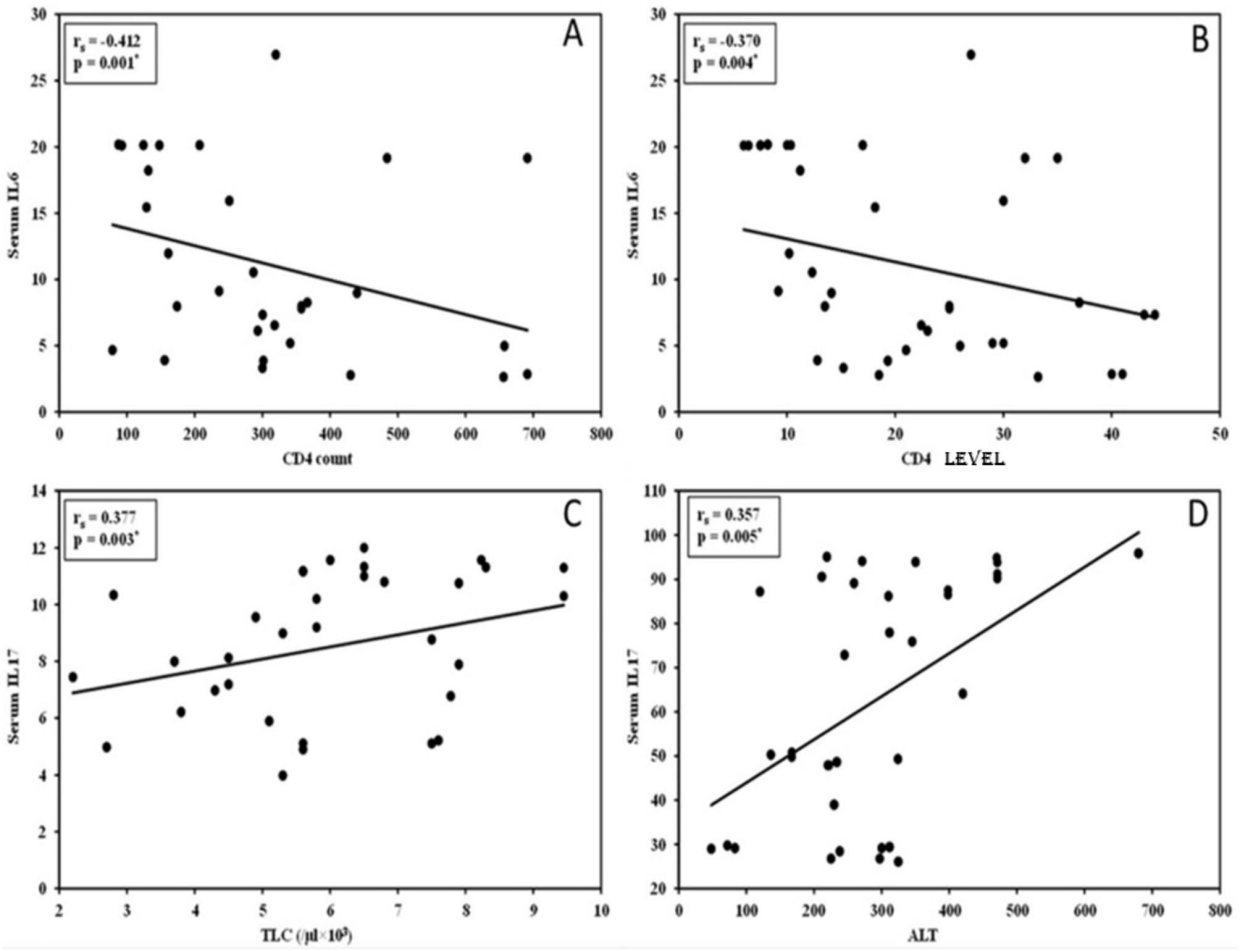
A. Correlation between serum IL-6 and CD4 count in group 2, B. Correlation between serum IL-6 and CD4 in group 2, C. Correlation between serum IL-17 and TLC (/µl × 10^3^) in group 2, D. Correlation between serum IL-17 and ALT in group 1.

Serum IL-6 levels significantly correlated negatively with CD4 levels and its count in group 2 (*p* = 0.004, 0.001), respectively. On the other hand, group 2’s total leucocyte count and group 1’s ALT levels showed a positive connection with serum IL-6 levels (*p* = 0.022, 0.005), respectively (Fig. 6).

In Table 7 after accounting for variance resulting from differences between groups, a univariate analysis was performed to evaluate the independent connection between possible factors and the interleukins under investigation. In order to prevent multi-co-linearity, the most pertinent variables were carefully chosen to be included in the regression model. This was done since several of these factors had strong correlations with one another and with the interleukins levels. With the exception of immunosuppressive medication regimen (cyclosporine) and serum IL-6 and IL-6 gene (CG + GG) levels (*P* = 0.011, 0.002, 0.028, respectively), which were later evaluated by multivariate analysis, none of the independent parameters interfered as a risk factor contributing to rejection.

**Table 7:** Univariate and multivariate Logistic regression analysis for the risk factors causing rejection (n = 60).

	Univariate		^#^Multivariate	
	*p*	B (LL–UL 95%C.I)	*p*	B (LL–UL 95%C.I)
Co morbidities				
Obesity	0.673	0.837 (0.366–1.915)		
Hypertension	0.702	0.864 (0.408–1.830)		
Renal disease	0.161	1.978 (0.761–5.141)		
Diabetes	0.252	0.643 (0.302–1.369)		
Original disease				
HCV.HCC	0.439	0.740 (0.346–1.586)		
HCV	0.465	1.308 (0.637–2.684)		
HBV	0.512	1.556 (0.416–5.819)		
Others	0.571	0.722 (0.234–2.224)		
Donor degree	0.228	1.343 (0.831–2169)		
Immunosuppressant regimen (Cyclosporine)	0.011*	2.591 (1.240–5.412)	0.134	4.700 (0.621–35.551)
Type of organism				
Staph aureus	1.000	1.0 (0.488–2.049)		
Klebsiella	0.854	0.935 (0.455–1.922)		
E.coli	0.132	0.444 (0.155–1.276)		
Pseudomonas	0.610	1.300 (0.475–3.560)		
Fungal infection				
Serum IL-17	0.989	–		
Serum IL-6	0.002*	1.601 (1.187–2.159)	0.006*	1.666 (1.160–2.395)
IL-17 gene (GA + AA)	0.803	1.133 (0.425–3.024)		
IL-6 gene (CG + GG)	0.028*	5.800 (1.213–27.728)	0.527	3.104 (0.093–104.122)

B: Unstandardized Coefficients, C.I: Confidence interval, LL: Lower limit, UL: Upper Limit, #: All variables with *p*<0.05 was included in the multivariate.

*Statistically significant at *p* ≤ 0.05.

The results of the multivariate logistic regression analysis for the genotypes (GG, CG) frequency of IL-6 genes revealed that the risk of having the CC gene was 5.8 times higher in liver post-transplant rejected patients than the non-rejected one (OR = 5.8,1.6), *p* = 0.007, 008 correspondingly (Table 8**)**.

**Table 8:** Multivariate Logistic regression analysis for serum IL-6 and IL-6 gene confirming their independency as risk factors causing rejection.

	Odd’s ratio		Adjust Odd’s ratio	
	*p*	OR (LL–UL 95% C.I)	*p*	OR (LL–UL 95% C.I)
Serum IL-6(pg/ml)	0.002*	1.601 (1.187–2.159)	0.008*	1.660 (1.144–2.410)
IL-6 gene (CG + GG)	0.028*	5.800 (1.213–27.728)	0.007*	16.984 (2.147–134.334)

OR: Odd**’s **ratio, Adjust Odd’s ratio by ALT, AST and Albumin, C.I: Confidence interval, LL: Lower limit, UL: Upper Limit.

*Statistically significant at *p* ≤ 0.05.

**Fig. 7:**
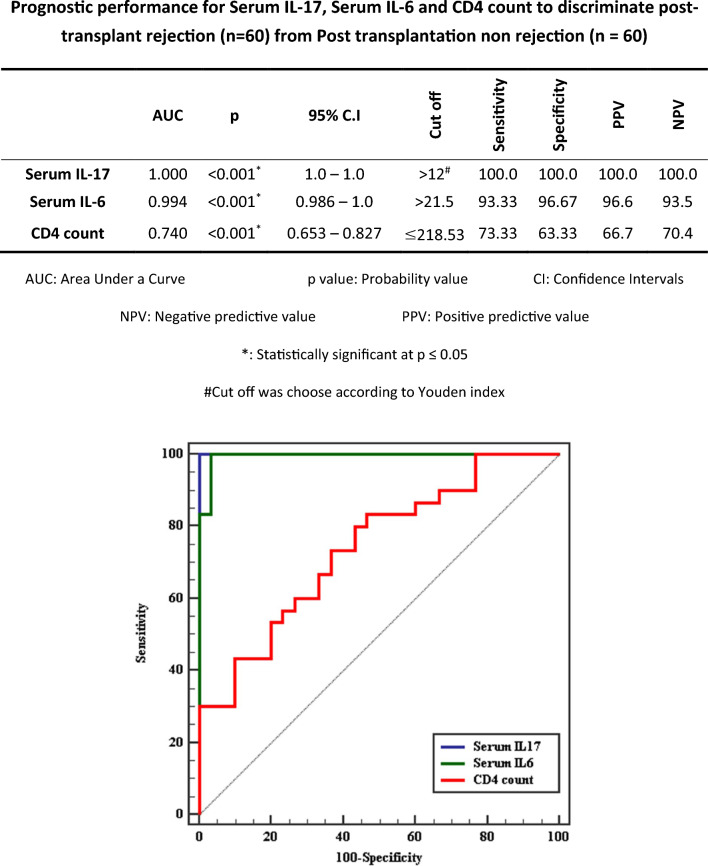
ROC curve for Serum IL-17, Serum IL-6 and CD4 count to discriminate post-transplant rejection (n = 60) from Post transplantation non rejection (n = 60).

To discriminate between positive and negative patients, data presented in Fig. 7 showed that, at the cutoff value > 12 pg/mL serum IL-17 levels will be more specific and sensitive than serum IL-6 and CD4 count to discriminate post-transplant rejection from Post transplantation non rejection cases with the specificity of 100% and sensitivity of 100% (Fig. 7**)**.

## Discussion

Because cytokines are strong immunomodulatory molecules with gene variations that affect inflammatory and immune responses, they can be used to assess the state of the liver microenvironment, influence the immune system’s reaction to rejection, or, on the other hand, mediate graft acceptance^3,18,19^.

Studies have looked at the effect of immunoregulatory gene polymorphisms as a risk factor on the results of transplants including the heart, lung, bone marrow, kidney, and liver. For instance, studies have linked kidney transplant rejection to cytokines such as IL-10, IL-4, IL-4R, and TNF-a, chemokines such as CCR2, CCR5, and MCP-1, adhesion molecules such as ICAM-1, and co-stimulatory molecules such as CTLA-4 gene polymer-phisms^20–22^. Furthermore, interindividual variability in the pharmacokinetics of immunosuppressant has been connected to cytokine gene polymorphisms in liver transplant patients^23^ or are predictive indicators of HCV severity^18^ and recurrence^24^, but not HBV infection^25^.

The primary outcome of this investigation was that there were significant differences in the genotype frequency (CC, CG, and GG) or alleles of IL-6 G-174C between the post-transplant rejection group and each of the post-transplant non-rejection and control groups (*p* = 0.011). However, there was no significant difference between the post-transplant non-rejection group and the control group in terms of genes and alleles (*p* = 0.71) and that concur with the 2011 Karimi et al.^25^. Who first reported that TGF-b and IL-4 gene polymorphisms were not statistically different in rejectors and non-rejectors, while IL-6 G-174C and IFN-c T? 874A variations were associated with acute rejection.

According to our research, a variety of inflammatory triggers cause the synthesis of IL-6 regulatory cytokine, which controls inflammatory reactions. The G allele is linked to higher IL-6 production and the IL-6-174C variant to decreased IL-6 production. The findings show a strong association between acute rejection and the GG genotype and the IL-6-174G allele. Although our data show a substantial correlation, three additional researches^26–28^ have established that there is no significant association between IL-6 G-174C SNP and acute liver rejection.

Because it can influence the innate and acquired immune response by activating neutrophils and antigen-presenting cells, IL-17 is present in several forms of rejection. Since IL-17 has a key role in triggering the production of cytokines and chemokines, such as IL-1β, IL-6, IL-8, and tumor necrosis factor-α (TNF-α)^29^, research on IL-17’s many roles in transplantation is becoming more and more interesting.

None of the GG, GA, and AA genotype frequencies or IL-17A alleles at location rs2275913 in the current investigation demonstrated significant differences between patient groups and the control group (*p* > 0.05). To enhance comprehension of the interleukins gene, the impact of genotypes on the production of interleukins under examination was determined for two patient groups, and the distribution of these interleukins was based on their serum level in the three genotype polymorphisms related to transplant rejection. Serum levels of IL-17A and IL-6 showed statistically significant variations between the two patient groups with distinct genotypes which agree with Millan et al. 2014 Who reported that Intracellular IFN-γ + and IL-2 + T-cells and IL17 may identify recipients at risk of acute rejection^30^. In patients with early allograft dysfunction, serum levels of IL-6 and IL-17 remained elevated in the first week following surgery chae et al.^31^. Sadly, there isn’t enough research on how IL-17 gene variants affect chronic rejection in humans, which makes comparing the data challenging.

Regarding bacterial infection, there were no significant differences between the patient groups and control group (*p* = 0.416) for any of the genotypes (GG, GA, and AA) frequency of IL-17A at a location rs2275913. There were notable variations in the frequency of IL-6 G-174C genotypes (CC, CG, and GG) between the patient and control groups. These changes might potentially be attributed to infection as a risk factor (*p* < 0.001).

Additionally, with fungal infection, there were significant differences between the patient groups and control group (*p* = 0.002, 0.01) in the genotypes (GG, GA, and AA) of IL-17A and the genotypes (CC, CG, and GG) of IL-6 G-174C.correspondingly.

Our findings were consistent with a research by Döring et al.^32^ that involved 61 paediatric patients who had received hematopoietic stem cell transplantation. According to the study, bacteremia was associated with a large increase in both IL-6 and IL-8, whereas ADV-viremia was associated with a significant increase in IL-6 alone. In addition, significant increases in IL-6, IL-8, and sIL-2R were seen in a sepsis. Post-transplant adverse events (GvHD and diarrhea from a viral infection, or VOD and liver GvHD) that shared comparable clinical symptoms may be differentiated from one another using cytokine analysis.

Boix et al. also reported the occurrence of opportunistic infection was significantly correlated with an imbalance between TH1, TH2 and TH17 cells in both liver and kidney transplant recipients^33^.

But in order to validate these findings and enable an appropriate treatment approach, larger patient cohort studies in a prospective setting will be carried out. These studies will focus on using characteristic cytokine patterns to identify post-transplant adverse events as early as the onset of fever with unknown origin or other initial clinical symptoms.

Serum IL-6 levels significantly correlated negatively with CD4 levels and its count in group 2 (*p* = 0.004, 0.001), respectively. On the other hand, a positive connection (*p* = 0.022, 0.005) was found between the levels of serum IL-6 and the total leucocyte count in group 2 and the ALT levels in group 1.

Regarding the effect of 16 SNPs chosen from the Th1 and Th2 cytokine genes on acute liver allograft rejection, a meta-analysis of seven similar investigations was conducted. According to this study, variations of TGF-b-869, IL-6-174, and 915 are not risk factors for acute rejection after liver transplantation; however the IL-10-1082 SNP is^34^. Immunosuppression and ethnicity have been suggested as potential contributing factors to discrepancies in the findings of several investigations^34^.

We discovered that serum IL-17 levels, with a 100% specificity and 100% sensitivity threshold, will be more sensitive and specific than serum IL-6 and CD4 count in differentiating post-transplant rejection from non-rejection patients, and that concur with the findings of Döring et al. (2015)^32^. Also Han etal found IL-6 and IL-17 levels are associated with early acute cellular rejection in LDLT patients^35^.

We observe two limitation in our study that we will need to take prospective clinical studies to assess these results and using larger sample size of patients.

## Conclusions

Proinflammatory cytokines may serve as potential markers for the early identification and distinction of adverse outcomes following transplantation, thus enabling timely and appropriate therapeutic intervention. It will take prospective clinical studies to assess these results.

## Data Availability

The datasets generated during and/or analysed during the current study are available from the corresponding author on reasonable request.

## Abbreviations

IL-6: Interleukin 6
IL-17: Interleukin 17
CD4: Cluster Differentiation 4
ESLD: End stage liver disease
LT: Liver transplantation
NLI: National Liver Institute
GvHD: Graft versus host rejection
VOD: Veno occlusion disease
LDLT: Living donor liver transplant
POD 4: Post-operative day 4
HSV: Human herpes simplex virus
VZV: Varicella zoster virus
EBV: Epstein Barr virus
CMV: Cyto-Megalo virus
ADV: Adeno virus
AST: Aspartate trans-aminase
ALT: Alanine trans-aminase
USA: United States of America
RAI: A-rejection activity index
PBS: Phosphate buffer saline
HB: Hemoglobin
WBCs: White blood counts
PLTs: Platelets
SNP: Single nucleotides polymorphism
DNA: De-oxy ribo nucleic acid
PCR: Polymerase chain reaction
